# Bioactive Compounds from Propolis on Bone Homeostasis: A Narrative Review

**DOI:** 10.3390/antiox14010081

**Published:** 2025-01-12

**Authors:** Vanessa Bertolucci, André Felipe Ninomiya, Giovanna Barbarini Longato, Luisa Oliveira Kaneko, Nilson Nonose, Pedro Paulo Menezes Scariot, Leonardo Henrique Dalcheco Messias

**Affiliations:** 1 Research Group on Technology Applied to Exercise Physiology—GTAFE, Health Sciences Postgraduate Program, São Francisco University, Bragança Paulista 12916-900, SP, Brazil; vanessa.bertolucci@mail.usf.edu.br (V.B.); nilson.nonose@usf.edu.br (N.N.);; 2 Research Laboratory in Molecular Pharmacology of Bioactive Compounds, São Francisco University, Bragança Paulista 12916-900, SP, Brazil; giovanna.longato@usf.edu.br

**Keywords:** oxidative stress, osteoclasts, osteoblasts, inflammation, propolis

## Abstract

This narrative review explores the potential effects of Propolis and its bioactive compounds on bone health. Propolis, a resinous product collected by bees, is renowned for its antimicrobial, anti-inflammatory, and antioxidant properties. Recent research emphasizes its positive role in osteogenesis, primarily through the modulation of osteoclast and osteoblast activity via molecular pathways. Key mechanisms include reducing inflammatory cytokines, protecting against oxidative stress, and upregulating growth factor essential for bone formation. While compounds such as Caffeic Acid Phenethyl Ester, Apigenin, Quercetin, and Ferulic Acid have been well-documented, emerging evidence points to the significant roles of less-studied compounds like Pinocembrin, Kaempferol, p-Coumaric acid, and Galangin. This review synthesizes the current literature, focusing on the mechanisms by which these bioactive compounds influence osteogenesis. Firstly, it explores the techniques for characterizing bioactive compounds presented in propolis, the chemogeographic variations in its composition, and the effects of both crude extracts and isolated compounds on bone tissue, offering a comprehensive analysis of recent findings across different experimental models. Further, it discusses the effects of Propolis compounds on bone health. In summary, these compounds modulate signaling pathways, including nuclear factor kappa beta, wingless-related integration site, mitogen-activated protein kinase, vascular endothelial growth factor, and reactive oxygen species. These pathways influence the receptor activator of nuclear factor kappa-β/receptor activator of nuclear factor kappa-β ligand/osteoprotegerin system, fostering bone cell differentiation. This regulation mitigates excessive osteoclast formation, stimulates osteoblast activity, and ultimately contributes to the restoration of bone homeostasis by maintaining a balanced bone remodeling process.

## 1. Introduction

Propolis, a resinous substance collected by bees from plant exudates, is widely used in traditional medicine due to its antimicrobial, anti-inflammatory, and antioxidant properties [1,2]. Propolis has a complex composition, with hundreds of identified compounds. Factors such as plant origin, geographical location, and seasonality influence its chemical composition [3,4].

Recent studies have shown that propolis and its bioactive compounds can positively influence osteogenesis by modulating the formation and activity of osteoclasts and osteoblasts through various molecular pathways [5,6]. These effects are primarily attributed to the antioxidant and anti-inflammatory properties of propolis, which help reduce the expression of inflammatory cytokines and protect bone cells from oxidative stress [7]. Additionally, propolis has been associated with increased expression of growth factors, such as Fibroblast Growth Factor 2 (FGF-2) and Vascular Endothelial Growth Factor (VEGF), which are crucial for angiogenesis and bone formation [8,9].

Although the literature on the effects of propolis on osteogenesis has grown exponentially in recent years, there remains a notable lack of narrative reviews discussing the differences among its bioactive compounds in this context, especially regarding recent evidence. Despite a wide range of compounds being studied, particular attention has been given to Caffeic Acid Phenethyl Ester, Apigenin, Quercetin, and Ferulic Acid. Systematic reviews have summarize the effects of these compounds on bone health [1,5,6,10]. However, recent evidence on less-studied compounds, such as Pinocembrin [11,12], Kaempferol [13,14,15], p-Coumaric acid [16,17,18], and Galangin [19,20]**,** also suggests significant effects on key bone-related processes. Researchers would benefit from a comprehensive discussion of published studies, especially considering the physiological mechanisms by which these compounds influence osteogenesis. Furthermore, there is substantial value in showcasing the beneficial effects of propolis and its bioactive compounds across different experimental models, such as in vivo and in vitro studies.

The primary objective of this narrative review is to summarize and discuss recent scientific literature on the effects of propolis bioactive compounds on bone health. Specifically, the review aims to identify and analyze the mechanisms of action for these bioactive compounds in osteogenesis. To this end, this review will first address propolis composition and chemogeographic variation, followed by techniques for characterizing its bioactive compounds. These approaches are particularly relevant given the recent scientific advancements that have focused on isolated compounds from propolis rather than the whole extract. Additionally, the effects of propolis extract on bone tissue and its primary actions on key signaling pathways will be presented. Finally, this review will present the effects of propolis’s active compounds on these pathways, along with other suggested mechanisms.

## 2. Materials and Methods

The databases PubMed, Scopus, and Google Scholar were used for the literature search. Keyword combinations, such as “propolis”, “bioactive compounds”, “bone”, “osteogenesis”, “osteoblasts”, “osteoclasts”, “caffeic acid phenethyl ester”, “apigenin”, “quercetin”, and “ferulic acid”, were employed. To summarize the most recent findings, only articles published within the last 10 years were considered. Original studies, systematic reviews, and meta-analyses that investigated the effects of propolis bioactive compounds on bone health, both in vitro and in vivo, were included. Studies not directly related to the topic, as well as opinion articles, editorials, reports, and conference abstracts, were excluded. The results of the selected studies were critically summarized and analyzed, highlighting key findings, limitations, and implications for future research.

## 3. Techniques for Characterizing Bioactive Compounds of Propolis Extract

### 3.1. General Considerations and Conventional Methods of Extraction

Propolis is abundant in bioactive compounds with significant therapeutic properties, each contributing uniquely to its biological activity [21]. Determining its botanical source is a crucial initial step before conducting bioactivity studies, as its chemical composition varies significantly depending on local flora, as well as geographic and climatic conditions [22,23]. This variability directly influences the bioactive compounds present in propolis, which may act synergistically, enhancing its beneficial effects [24]. Propolis’s raw materials cannot be used directly and must be extracted and purified [25] to remove inert substances and to retain polyphenols, which are generally considered the most important components of propolis [26].

The biochemical composition and bioavailability of propolis extracts are significantly influenced by the solvent polarity and the extraction method used [2,21]. The traditional maceration method, which is widely employed, involves separating the active compounds after extraction [27]. Common solvents include water and ethanol; however, due to the low solubility of propolis in water, ethanolic extracts typically contain higher concentrations of total polyphenols and lower wax content compared to aqueous extracts [24,26,28,29]. Furthermore, using a high-ethanol solvent produces extracts with enhanced antioxidant activity [21,30].

Ethanolic extraction is particularly effective due to its ability to solubilize a broader range of phenolic compounds [25,26]. However, ethanol can cause skin irritation and is unsuitable for children, pregnant women, and individuals allergic to ethanol, among other sensitive groups [2,31]. To address these limitations, alternative solvents, such as propylene glycol, polyethylene glycol, glycerol, and vegetable oils, have been considered. These options not only provide effective extraction, but are also pharmaceutically safe and non-toxic, offering viable alternatives to traditional alcoholic solutions [28,32].

The extraction of bioactive compounds from propolis remains the focus of studies aimed at developing more efficient and sustainable methods [30,33]. Several innovative techniques have emerged. Ultrasonic extraction enables the rapid extraction of flavonoids at low temperatures with high yields [33]. The combination of ultrasound and microwave processing offers quick and efficient extraction under mild conditions [30]. Ultra-high-pressure extraction enhances solvent penetration and mass transfer, significantly increasing the extraction rate [34]. Meanwhile, extraction using supercritical fluids, particularly CO2, preserves thermosensitive compounds and leaves no residues, making it a clean and sustainable technology for the chemical, pharmaceutical, and food industries [24,31,35].

### 3.2. Advanced Analytical Techniques

The bioactive compounds present in propolis may act synergistically, enhancing their positive effects on bone tissue [24]. However, to maximize these effects and improve bioavailability, it is essential to characterize and isolate these compounds. The process of characterizing and isolating bioactive compounds from propolis allows for an accurate assessment of their pharmacological properties, including antioxidant, anti-inflammatory, and anticancer activities as well as toxicity, bioavailability, and bioabsorption [36]. Identifying and quantifying these compounds are crucial for understanding their mechanisms of action and determining effective and safe therapeutic doses [36,37].

Advanced techniques are essential for a precise and specific characterization of the chemical composition of the propolis complex matrix, composed of hundreds of compounds and a wide variety of polyphenols, flavonoids, terpenes, and other secondary metabolites [4,24]. High-resolution analytical methods, such as High-Performance Liquid Chromatography (HPLC), Ultra-High-Performance Liquid Chromatography coupled with Tandem Mass Spectrometry (UHPLC-QqQ-MS/MS), Liquid Chromatography coupled with Electrospray Ionization Tandem Mass Spectrometry (LC-ESI-MS/MS), and Time-of-Flight Mass Spectrometry (QTOF-MS), enable the separation, identification, and quantification of these compounds with high sensitivity and specificity [26,37]. Moreover, these techniques allow for the discovery of new bioactive compounds, contributing to the elucidation of its therapeutic potential and the development of innovative pharmaceutical and nutraceutical products [38,39].

HPLC is widely utilized for the separation, identification, and quantification of phenolic compounds and flavonoids in propolis [26]. The ability to couple HPLC with various detectors, such as UV-visible detectors and diode array detectors (HPLC-UV, HPLC-DAD), as well as gas chromatography coupled with mass spectrometry (GC-MS), is extensively used for the detection and analysis of flavonoids and phenolic compounds present in this resinous substance [40,41,42]. No less important, UHPLC-QqQ-MS/MS is a highly sensitive technique used to determine phenolic compounds based on the monitoring of specific molecular ions and their corresponding ion transitions [43,44].

LC-ESI-MS/MS combines chromatographic separation with mass spectrometric detection, allowing for the precise identification of bioactive compounds. Electrospray ionization facilitates the ionization of compounds in the liquid phase, which are then separated and detected with high sensitivity and specificity. This technique is particularly useful for analyzing phenolic compounds and flavonoids in propolis extracts [26,45]. QTOF-MS also offers high resolution and accuracy in determining the molecular mass of compounds. This technique is employed for the identification of novel bioactive compounds in propolis, enabling specific characterization of their chemical structure. QTOF-MS is often combined with liquid chromatography (LC-QTOF-MS) for analyzing complex propolis extracts [45,46].

The use of advanced techniques is crucial for exploring the chemical complexity of propolis and identifying the compounds responsible for its biological properties, driving research and the development of applications for this important natural product [26].

## 4. Propolis Composition and Chemogeographic Variation

Propolis is a resinous substance that bees collect from plant exudates, including flowers, leaf buds, resins, gums, and mucilages. This material is enriched with bee saliva, which contains enzymes such as β-glucosidase [23], along with other bee-specific salivary enzymes [6,23]. Hundreds of components have been identified in propolis. Constituents in most propolis samples include phenolic acids, prenylated benzophenones, flavonoid glycosides, flavonoid aglycones and their esters, volatile organic compounds and their esters, phenolics, sesquiterpenes, quinones, coumarins, steroids, aldehydes, alcohols, ketones, and amino acids [6,47]. Propolis is also a rich source of essential elements like magnesium, nickel, calcium, iron, zinc, cesium, manganese, silver, copper, aluminum, vanadium, amino acids, and vitamins B, C, and E [5,6,38,47,48,49,50]. In the plant kingdom, phenolic compounds—particularly flavonoids—typically occur as glycosides in subclasses like flavanones, flavones, flavonols, and dihydroflavonols. However, in propolis, these compounds predominantly exist as aglycones due to the action of glycosidase enzymes produced by bees [38].

The botanical origin of the resin is fundamental in defining a specific type of propolis, with climate and geography playing key roles in shaping the characteristics of biomes and their flora [51]. The chemogeographical variation of propolis reflects the botanical diversity across different global regions. This variation leads to unique phytochemical profiles and specific biological properties [47]. Therefore, determining the botanical source of propolis is a crucial preliminary step before conducting bioactivity studies, as its chemical composition varies significantly depending on local flora, geographical conditions, and climate [22,23]. Consequently, investigating the chemical composition of propolis and its plant sources is essential for understanding its geographical diversity, making this topic particularly relevant [52]. Propolis samples from temperate zones (e.g., West Asia, North Africa, Europe, North America, parts of Argentina, and New Zealand) have similar compositions and are rich in flavonoids and phenolic acid esters [47,53].

Chinese propolis is primarily sourced from poplar (*Populus* sp.) and is rich in phenolic acids and flavonoids, compounds that contribute to its notable antioxidant and anti-inflammatory properties [39,54]. The presence of the bioactive components, such as Galangin and chrysin, underscores its pharmacological potential in combating oxidative stress and associated diseases [3]. European propolis, also derived from *Populus* spp., is characterized by its high levels of caffeic acid, ferulic acid, flavonoids, and aromatic esters [55]. Propolis collected from mountainous regions in Russia, Switzerland, and Italy is characterized by phenolic glycerides, including dicoumaroyl acetyl-, diferuloyl acetyl-, feruloyl coumaroyl acetyl-, and caffeoyl coumaroyl acetyl glycerol. These compounds derive from *Populus tremula*, which grows in cooler climates and at higher altitudes [47,56]. In contrast, Brazilian green propolis is derived from *Baccharis dracunculifolia* and is distinguished by its abundance of prenylated phenylpropanoids, being Artepillin C the most abundant. It also contains caffeic acids, cinnamic acids, p-coumaric acid, ferulic acid, and their derivatives, alongside diterpenes and flavonoids [51,57,58,59]. Other types of propolis found in Brazil include red and brown propolis. Derived from *Dalbergia ecastophyllum*, a mangrove-associated shrub, red propolis contains distinctive isoflavones and neoflavonoids, such as formononetin. It exhibits antimicrobial, wound-healing, and anticancer properties, making it a valuable type for medicinal use [51,57,58,59]. Brown propolis originates from a variety of plants, including *Eucalyptus* spp. and *Baccharis* spp. Its chemical composition includes flavonoids, phenolic acids, and aromatic esters. Brown propolis is the most commonly available type and is widely used in cosmetics and dietary supplements [60]. Russian birch propolis is sourced from *Betula verrucosa*, with key compounds being flavones and flavonols, distinguishing it from the poplar type [61]. Venezuelan and Cuban propolis originates from *Clusia minor* and *Clusia rosea* species and is characterized by polyisoprenylated benzophenones [59]. Chilean propolis is known to contain various classes of compounds, including phenylpropanes, benzaldehydes, dihydrobenzofurans, benzopyrans, and lignans. The botanical origin of this propolis is associated with species such as Eucalyptus and Ricinus, among other native Chilean species [47,62]. Propolis samples from the Pacific, obtained in Taiwan and Japan (Okinawa), predominantly contain prenylated flavanones linked to the botanical source *Macaranga tanarius* [63].

In Chinese propolis, several important flavonoids and phenolic compounds have been identified, like pinobanksin and phenolic acids, including caffeic acid, as well as pinocembrin and chrysin [26,64]. Indonesian propolis was found to contain petunidin and gingerol C, while unique compounds, such as dumasin and myristicin, were identified in New Zealand samples [41,65]. Brazilian propolis revealed red propolis markers like retusapurpurin A and green propolis phenolics, such as artepillin C [37,66].

Propolis from the Canary Islands, Colombia, and Costa Rica showed distinctive compounds like furofuran lignans, chrysosplenol-O-methyl-ether, and nemorosone, respectively [67,68,69]. In regions like Kazakhstan, India, and Turkey, analyses highlighted flavonoids, polyphenolic compounds, and specific markers like lasiocarpins [42,49]. Additionally, studies from Mexico and Nigeria identified anthraquinones and flavonoid derivatives, reflecting the diverse phytochemical profiles of global propolis samples [4,70].

## 5. Mechanism of the Effect of Propolis on Osteogenesis and Osteoclastogenesis

Propolis has been proposed as a natural compound with anabolic (stimulating bone formation) and anticatabolic (reducing reabsorption) benefits [5,6,71]. In vitro and in vivo model studies demonstrated significant effects on osteogenesis by influencing the formation of both osteoblasts and osteoclasts through various molecular pathways [5,71]. It enhances osteoblast differentiation and activity, increasing the expression of key markers. These effects are mediated by its antioxidant and anti-inflammatory properties, which regulate osteoclast differentiation and maturation while promoting osteoblast proliferation and mineralization [1,10,57].

In osteoblastogenesis, propolis modulates the expression of critical transcription factors, such as Runt-related transcription factor 2 (RUNX2) and Osterix. RUNX2 plays a pivotal role in the differentiation of mesenchymal cells into osteoprogenitors, whereas Osterix is essential for the final maturation of osteoblasts [5,6]. Propolis also increases alkaline phosphatase (ALP) activity, which is vital for bone formation and mineralization [71,72]. Additionally, propolis impacts osteoclastogenesis by modulating the Receptor Activator of Nuclear Factor Kappa-β Ligand/Receptor Activator of Nuclear Factor Kappa-B/Osteoprotegerin (RANKL/RANK/OPG) pathway. Specifically, it promotes the binding of OPG to RANKL, thereby inhibiting its interaction with RANK on osteoclast precursors. This action reduces osteoclast formation and bone resorption, contributing to improved bone health [5,6,73].

### 5.1. Propolis Promotes Bone Formation and Prevents Bone Resorption via Antioxidant and Anti-Inflammatory Effect

Oxidative stress, characterized by an imbalance between the production of reactive oxygen species (ROS) or reactive nitrogen species (RNS) and endogenous antioxidant defenses, plays a critical role in the pathogenesis of various diseases, including bone disorders [5,74]. When the degree of oxidation exceeds the clearance of oxides, an imbalance between the oxidative and antioxidant systems occurs, leading to tissue damage [75], adversely impacting bone homeostasis.

During the inflammatory response, leukocytes and mast cells accumulate in the damaged areas. This process is characterized by an increase in oxygen uptake, resulting in a greater generation and release of ROS at the injury site [76]. Although complex, the inflammatory pathway can be triggered by ROS, and its reduction is crucial for mitigating inflammation [22,76]. Propolis exerts an inhibitory effect on neutrophil migration, thereby reducing both acute and chronic inflammatory responses [6,71]**.**

Pro-inflammatory cytokines exacerbate the inflammatory response, facilitating bone resorption and impairing bone formation. This process can promote osteoclastogenesis and bone resorption while simultaneously inhibiting osteoblastogenesis and bone formation, accelerating bone loss and significantly increasing the risk of fractures [5,71]. Propolis has been demonstrated to decrease the expression of pro-inflammatory cytokines, such as IL-12, IL-6, GM-CSF, and IFN-γ, while promoting an increase in regulatory cytokines, including IL-4, IL-10, and TGF-β, as well as Tumor Necrosis Factor-alpha (TNF-α) and Interleukin-1 beta (IL-1β), which are known to promote osteoclastogenesis [5,6]. This anti-inflammatory modulation is crucial, as chronic inflammation and oxidative stress are key drivers of bone homeostasis imbalance, resulting in bone loss [21]. Furthermore, propolis has the ability to inhibit the synthesis of prostaglandin E2 and the inducible expression of cyclooxygenase-2 [1,7]. By reducing inflammatory cytokines, propolis creates a more favorable environment for bone formation, decreasing osteoclast activity and enhancing osteoblast function [5,71].

The Nuclear factor erythroid 2-related factor 2/Kelch like ECH-Associated Protein 1 signaling pathway (NRF2/KEAP1) emerges as a promising target for the modulation of cellular processes, such as inflammation and osteogenesis. Studies highlight that natural compounds can activate NRF2 by increasing the expression of the antioxidant enzyme HO-1, which inhibits pro-inflammatory NF-κβ signaling and reduces levels of inflammatory cytokines. These mechanisms position the ROS/KEAP1/NRF2 signaling axis as central in the regulation of oxidative stress and inflammatory response, regulating osteoclast differentiation and function, with NRF2 activation leading to an increased expression of antioxidant enzymes, a reduction in ROS, and an attenuation of osteoclastogenesis [77,78,79,80].

Estrogen plays a crucial role in bone metabolism by inhibiting bone resorption and promoting bone formation. Flavonoids, which are compounds with estrogen-like properties, are often used as substitutes for estrogen and may help protect against bone loss associated with menopause [81]. Postmenopausal bone loss is associated with increased osteolytic activity from osteoclasts, due to reduced estrogenic activity mediated by the ERα receptor. Estrogen, through ERα, suppresses the production of the NF-κβ receptor activator (RANKL) ligand by bone lining cells; its absence, therefore, leads to an overproduction of RANKL and an intensification of osteoclastic activity. Activated T-cell nuclear factor c1 (NFATc1) plays a central role in the maturation and function of osteoclasts. In this context, NRF2 activation emerges as a relevant mechanism, as it negatively regulates the expression and activity of NFATc1 in osteoclasts. Thus, the NRF2 pathway represents a promising therapeutic target to preserve bone mass in conditions such as estrogen deficiency, glucocorticoid treatment, chronic inflammation, and senile osteoporosis [82].

### 5.2. Angiogenesis and Osteogenesis

The interaction between osteogenesis and angiogenesis is crucial for bone homeostasis and regeneration. Bone and endothelial cells communicate through growth factors, establishing a complex and dynamic bidirectional relationship. Vascular endothelial growth factor (VEGF) stands out as the primary regulator of physiological and pathological angiogenesis in this process [8,9,83]. VEGF promotes angiogenesis through the endogenous production of ROS and the migration of endothelial cells. The interaction between oxidative stress and angiogenesis is largely mediated by VEGF signaling. In the osteogenic environment, both osteoprogenitor and inflammatory cells express VEGF [75,84]. However, a deficiency in angiogenesis impairs ossification and delays bone healing, while excessive angiogenesis is associated with diseases such as osteosarcoma [75].

Flavonoids present in propolis stimulate the expression of fibroblast growth factor-2 (FGF-2), VEGF-A, Osterix, RUNX2, and ALP [6,85]. This promotes the formation of new blood vessels, delivering essential nutrients for the proliferation and differentiation of osteoblasts [38]. The stimulation of these growth factors by propolis notably contributes to improved bone mineral density and the structural integrity of the skeleton [5,86].

Bioactive compounds found in propolis, such as cinnamic acid, have been shown to increase ALP activity and calcium levels, facilitating bone formation while inhibiting NF-κβ and TNF-α. Additionally, the phenolic compounds in propolis possess the capacity to promote bone regeneration, potentially due to their regulatory effects on the accumulation of collagen (types I and III) and their beneficial impact on the deposition of chondroitin sulfate and hyaluronic acid at sites of tissue injury [6].

### 5.3. Intracellular Signaling Pathways Affected by Propolis

Propolis has been shown to significantly influence the mitogen-activated protein kinase (MAPK) signaling pathway, which is crucial for the formation and maintenance of bone tissue. Studies indicate that propolis can activate the Extracellular signal-Regulated Kinase (ERK) and c-Jun N-terminal Kinase (JNK) cascades, which are essential for osteoblast differentiation and bone formation [1,38]. The activation of these pathways by growth factors and cytokines is fundamental to osteoblastogenesis; conversely, inhibition of the MAPK pathway can lead to defects in bone formation [33,38,70].

Propolis indeed negatively regulates the activity of transcription factors like NF-κβ [1]. The activation of the NF-κβ pathway by stimuli such as TNF-α and IL-1β is essential for the differentiation of osteoclasts and the expression of osteogenic transcription factors [7]. However, activation of NF-κβ increases bone resorption by osteoclasts and may inhibit bone formation by osteoblasts. By regulating this pathway activity, propolis helps mitigate these effects [1,10].

**Figure 1:**
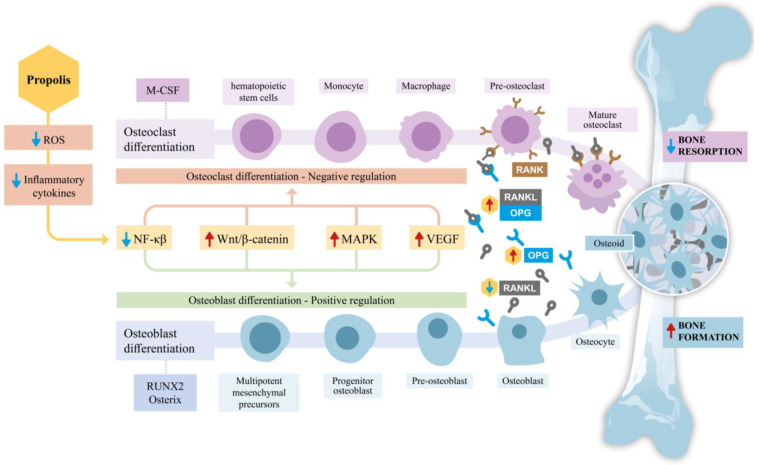
Schematic representation of the effects of propolis on bone remodeling, emphasizing osteoblast and osteoclast differentiation. Propolis reduces reactive oxygen species (ROS) and inflammatory cytokines, thereby influencing key signaling pathways involved in bone metabolism. By downregulating nuclear factor kappa β (NF-κβ) activity and modulating critical pathways such as Wingless/Integrated β-catenin (Wnt/β-catenin), mitogen-activated protein kinase (MAPK), and Vascular endothelial growth factor (VEGF), propolis inhibits osteoclast differentiation through the Receptor Activator of Nuclear Factor Kappa-β Ligand/Receptor Activator of Nuclear Factor Kappa-B/Osteoprotegerin (RANK/RANKL/OPG) axis, effectively reducing bone resorption. Simultaneously, it promotes osteoblast differentiation by upregulating transcription factors, such as Runt-related transcription factor 2 (RUNX2) and Osterix, thereby enhancing osteoid production and bone formation. Together, these effects shift the balance towards increased bone formation and reduced bone resorption, highlighting propolis’s potential therapeutic role in supporting bone health.

The Wingless/Integrated (Wnt) signaling pathway involves ligands that bind to specific cell surface receptors, such as Frizzled proteins and Low-Density Lipoprotein Receptor-Related Proteins (LRPs). In the presence of Wnt signaling, β-catenin accumulates in the cytosol, translocates to the nucleus and forms a complex with transcription factors to activate target genes, thereby modulating the differentiation of precursor cells into osteoblasts [87]. While β-catenin is associated with promoting tumorigenesis, cancer progression, and invasion [88], propolis may contribute to the stabilization of β-catenin. This stabilization is linked to a reduction in osteoclastic activity and the inhibition of apoptosis in osteoblasts and osteocytes, favoring the formation and maintenance of bone tissue [5,89]. Figure 1 summarizes the effects discussed thus far regarding the impact of propolis on signaling pathways, with a particular focus on osteoblasts and osteoclasts.

## 6. Effects of Bioactive Compounds in Propolis Extracts on Bone Health

Although propolis has shown beneficial effects on bone health, a broader range of studies is necessary to assess the safety, efficacy, and mechanisms of action of isolated compounds [4,21,66]. The following sections discuss evidence on this topic.

### 6.1. Caffeic Acid Phenethyl Ester

The caffeic acid phenethyl ester, commonly known as CAPE (Caffeic Acid Phenethyl Ester), is a bioactive compound of significant scientific interest. With a C_17_H_16_O_4_ chemical formula and a molecular weight of 284.31 g/mol, CAPE is primarily found in temperate regions. CAPE is a phenylpropanoid naturally found in propolis [90,91], which is sourced from plants like the genus *Populus*, which includes several species of poplars [56,64].

CAPE is a type of polyphenol characterized by hydroxyl groups on the catechol ring [90,92]. These hydroxyl groups play a critical role in various biological functions [91]. CAPE is noted for its low toxicity, showing no adverse effects or reduction in the viability of normal cells [93]. Its elimination half-life is dose-independent, ranging from 21.2 to 26.7 min. In vitro stability studies reveal that CAPE hydrolyzes into caffeic acid after 6 h in rat plasma [93,94]. Pharmacodynamics and pharmacokinetics vary between murine and human organisms; for example, CAPE is more stable in human plasma than in rat plasma, likely due to enzymatic differences [90]. CAPE exhibits a range of activities, including antibacterial, antidiabetic, antioxidant, anti-inflammatory, antineoplastic, and cardioprotective activities [95].

This compound acts as a specific inhibitor of NF-κβ, suppressing inflammation-related biological processes [96,97]. CAPE inhibits NF-κβ activation, reducing the production of inflammatory cytokines, including TNF-α, interleukins-1β, 6, and 8, as well as the expression of cyclooxygenase-2 (COX-2). Furthermore, it significantly inhibits osteoclast formation and differentiation, while inducing apoptosis in these cells in primary cell models [90,98,99].

CAPE modulates the RANKL/RANK/OPG signaling pathway, inhibiting RANKL expression and increasing OPG expression, thereby reducing bone resorption [100]. It promotes the expression of the transcription factor RUNX2, enhancing bone formation and improving bone mineral density [99,101,102]. Furthermore, it possesses antioxidant properties that protect bone cells from oxidative stress, a major factor that can compromise bone remodeling. Studies in osteoporosis models have shown CAPE’s ability to restore oxidative balance in bone tissues [95]. CAPE also modulates MAPK pathways, although its specific effects depend on cell type and the concentration used [33,98].

In murine macrophages, CAPE demonstrated a significant anti-osteoclastic effect. According to Kwon et al. (2018), this was attributed to the suppression of superoxide anion production, mediated by the prevention of active Nox1 complex formation. CAPE attenuated the translocation of p47Phox to the cell membrane, a mechanism similar to that observed for apocynin, a Nox1 inhibitor. This led to reduced superoxide anion levels, essential for RANKL-induced osteoclast differentiation [92].

Potent anti-inflammatory and antioxidant effects in vivo were also shown for this compound, protecting against osteoarthritis (OA) progression. Sun et al. (2022) investigated the effects of CAPE on IL-1β-stimulated chondrocytes in vitro and surgically induced OA models in rats. CAPE reduced the expression of inducible nitric oxide synthase (NO) and cyclooxygenase-2 (COX-2), along with a decrease in extracellular secretion of NO and prostaglandin E2. Additionally, it also attenuated extracellular matrix degradation and NF-κβ signaling. In vivo, CAPE preserved cartilage integrity and slowed OA progression, suggesting its potential as a therapeutic agent for OA prevention or treatment. CAPE activates the NRF2/HO-1 signaling pathway in human chondrocytes stimulated by IL-1β, increasing NRF2 and HO-1 expression in a concentration-dependent manner. Western blot and immunofluorescence analyses showed greater nuclear translocation of NRF2 after pretreatment with CAPE, indicating that its anti-inflammatory effects are mediated by the activation of this pathway [103].

CAPE has also shown protective effects against glucocorticoid-induced osteoporosis. Tolba et al. (2017) observed that CAPE modulated the RANKL/RANK/OPG pathway, restored oxidative balance, and reduced bone resorption by increasing OPG expression, thereby offering protection against glucocorticoid-induced osteoporosis. Similarly, Kizildag et al. (2019) reported that CAPE decreased bone resorption in rats with endotoxin-induced periodontitis by modulating the RANKL/RANK/OPG pathway and reducing reactive oxygen species (ROS) levels [99,100].

In vitro studies have highlighted CAPE’s ability to stimulate osteogenesis. Santos et al. (2021) reported that low concentrations enhanced RUNX2 expression and modulated the Wnt/β-catenin pathway, promoting bone formation and improving bone mineral density. Experimental models conducted by Acikan et al. (2022) demonstrated that CAPE improved bone fracture healing by increasing bone formation and decreasing resorption. Similarly, Narmada et al. (2021) showed that CAPE reduced inflammation and significantly elevated both osteoblast numbers and fibroblast growth factor-2 (FGF-2) expression during experimental tooth movement in rats. Xu et al. (2023) explored its effect on titanium particle-induced bone loss in a mouse model, finding a significant suppression of bone degradation via a reduction in the RANKL/OPG ratio and osteoclastogenesis. Additionally, the treatment had downregulated the expression and secretion of pro-inflammatory cytokines, including IL-6, IL-1β, and TNF-α, indicating its potential to prevent titanium particle-induced bone loss. Zawawi et al. (2015) examined CAPE’s effects in a murine calvarial model of polyethylene particle-induced osteolysis, finding that a low-dose administration significantly decreased surface and volumetric bone resorption. This effect was attributed to the inhibition of osteoclast activity and a reduced expression of Nuclear Factor of Activated T-cells, Cytoplasmic 1 and NF-κβ [97,101,102,104,105].

### 6.2. Apigenin

Apigenin (API) is a bioactive flavonoid compound derived from propolis [23], which is sourced from the genus *Apium* of the Apiaceae family. It is found in various vegetables and fruits particularly in warm tropical regions. Its chemical formula is C_15_H_10_O_5_ with a molecular weight of 270.24 g/mol. Structurally, apigenin consists of two aromatic rings (A and B) connected by a 3-carbon bridge (C ring) [106]. It is primarily present in its glycosylated form, which is more water-soluble than unmodified apigenin, and may offer higher bioavailability [107].

API exhibits potent antioxidant activity closely linked to its structural characteristics, especially the number of hydroxyl groups, which enable hydrogen atom donation to neutralize free radicals [106,108]. Its antioxidant mechanisms include the inhibition of oxidant enzymes, such as xanthine oxidase (XO), COX-2, and NO. Furthermore, API scavenges reactive oxygen and nitrogen species (ROS/RNS), interacts with redox signaling pathways, chelates transition metals, and enhances the levels of both enzymatic and non-enzymatic antioxidants [106,109]. It also exhibits a wide range of pharmacological activities, including antitumor, anti-inflammatory, and cardioprotective effects. However, its clinical and therapeutic application is limited by its low bioavailability, attributed to its poor water solubility, low intestinal absorption, and rapid metabolism [110,111]**.**

API has a bidirectional regulatory effect on bone metabolism, promoting osteogenic differentiation while inhibiting osteoclastogenesis, suggesting that treatment with this compound may be a novel and promising therapeutic strategy for osteoporosis and osteoarthritis [109,112]. Notably, it positively modulates the Wnt/β-catenin pathway, which is crucial for osteoblast differentiation, resulting in enhanced osteogenic activity. This indicates that API could be a potential therapeutic candidate for bone fractures [113]. While it significantly inhibits osteoclastogenesis and osteoclast function, information regarding its effects on overall bone metabolism remains limited [114]. It promotes osteogenesis by influencing the differentiation of mesenchymal progenitor cells into osteoblasts. Equally important, it has been suggested that it also increases the expression of RUNX2 [115].

This compound significantly reduces the secretion of various pro-inflammatory cytokines, specifically TNF-α, IL-1β, IL-6, IL-10, and IL-12 [112,116]. Its anti-inflammatory and antioxidant effects are particularly relevant in the context of osteoporotic osteoarthritis. A comparative study by Tantowi et al. (2020) between an API-rich glycoside extract and diclofenac in an osteoporotic osteoarthritis rat model showed that API significantly reduced cartilage erosion, bone loss, catabolic cartilage changes, and inflammation. It also decreased serum biomarkers of osteoporotic osteoarthritis, NF-κβ expression, and matrix metalloproteinase-13 activity, with effects comparable to diclofenac. This suggests that apigenin glycosides are effective in preventing osteoporotic osteoarthritis [117].

API promotes osteogenic differentiation and accelerates bone fracture healing. Pan et al. (2021) investigated its ability to enhance the osteogenic differentiation of human mesenchymal stem cells. They found that this compound increases β-catenin expression and several downstream target genes of the Wnt/β-catenin signaling pathway, thereby activating this signaling cascade. In a rat femur fracture model, API improved new bone formation and accelerated fracture consolidation. These findings indicate apigenin’s potential as a therapeutic agent for bone fracture repair [113].

### 6.3. Quercetin

Quercetin (QCT) is found in the propolis of different species of bees [23], it is sourced from vegetables and fruits, such as apples, onions, berries, and cabbages, among others. Its chemical formula is C_15_H_10_O_7_ with a molecular weight of 302.24 g/mol. QCT consists of 2 benzene rings connected by a 3-carbon chain that forms a closed pyran ring. Glycosylation can occur in any hydroxyl group, producing various forms of quercetin glycoside. Its biological activity is attributed to these active phenolic hydroxyl groups and double bonds [118,119].

In terms of excretion, the oral clearance of QCT is rapid, with a short half-life in the blood [120]. Despite its health benefits, this compound is poorly soluble in water and has low oral bioavailability, limiting its therapeutic use. To address this, researchers have developed ways to enhance its bioavailability, including new formulations and structural modifications, such as glycoside-sulfate conjugates and derivatives [121].

QCT exerts beneficial effects on bone health, primarily by inhibiting osteoclast formation and activity [122]. It acts by modulating the expression of RANKL, increasing the expression of OPG, thereby preventing its interaction with RANK on osteoclasts, resulting in decreased bone resorption and the promotion of bone formation [123]. It promotes the differentiation of osteoblasts through the activation of the MAPK signaling pathway, increasing the expression of osteogenic proteins, such as RUNX2 and Osterix [122,123]. QCT increases the concentrations of ALP [124] and promotes the differentiation and activity of osteoblasts while reducing the differentiation and activity of osteoclasts through the Wnt/β-catenin, BMP/RUNX2, OPG/RANKL/RANK, ERK/JNK, oxidative stress, apoptosis, and transcription factor pathways [122].

This compound reduces the expression of osteoclast-related markers by inhibiting the NF-κβ activation, thereby suppressing osteoclast maturation and bone resorption [120]. QCT reduces inflammation by decreasing TNF-α, IL-1β, and IL-6 levels, while increasing anti-inflammatory cytokines like IL-10, creating a more favorable environment for osteogenesis [123]. QCT plays modulatory, biphasic, and regulatory roles on inflammation and exhibits strong anti-inflammatory capabilities, as demonstrated in diverse cell types in animal and human models [125].

In the process of cellular differentiation and osteogenesis, Pang et al. (2018) demonstrated that quercetin significantly increases the proliferation of bone marrow-derived mesenchymal stem cells (BMSCs), enhances ALP activity, and promotes mineralization, further inducing the expression of bone morphogenetic protein 2 (BMP2) and RUNX2, among others, through an estrogen receptor-mediated pathway. The use of the estrogen receptor inhibitor blocked these effects, indicating that the action of QCT is estrogen receptor-dependent [126]. Similarly, its effects on the differentiation of human mesenchymal stem cells into osteoblasts and adipocytes were investigated. Low concentrations of QCT may protect or promote bone formation by inhibiting osteoclast formation without inducing mesenchymal stem cells differentiation into adipocytes, while high concentrations may inhibit osteoblastogenesis and increase adipogenesis [127]. This compound enhances BMSC proliferation and osteogenic differentiation by indirectly activating the Wnt/β-catenin pathway. It increases BMSC proliferation, ALP activity, and the expression of osteogenic markers, such as BMP2, osteocalcin, and RUNX2 [128].

The effects of QCT extends to both healthy and tumorous osteoblasts. In healthy osteoblasts, QCT induces proliferation, migration, adhesion, and differentiation via the Protein Kinase B/Glycogen Synthase Kinase 3 Beta/β-catenin pathway. However, in tumorous osteoblasts, QCT induces apoptosis by activating ERK and inhibiting the Protein Kinase B/Bcl-2 Associated Death Promoter pathway. Low concentrations of QCT stimulated osteoblastogenesis without affecting the growth of tumorous osteoblasts, while high concentrations were effective against the tumorous cells [129]. In the context of iron overload-induced bone loss, QCT enhanced ALP activity, promoted the formation of mineralized bone nodules and positively regulated the expression of RUNX2 and Osterix. Additionally, it activated the nuclear factor erythroid 2-related factor 2 signaling pathway, thereby mitigating the damage induced by oxidative stress [130].

The pharmacological mechanisms of QCT against osteoporosis have been validated through in silico and in vivo approaches. This compound enhances osteoblastic differentiation and activity while simultaneously reducing osteoclastic differentiation and activity [131]. In combination with Dasatinib, it improved the bone tissue microenvironment by targeting and reducing senescent cells, mitigating the senescence-associated secretory phenotype, and restoring mesenchymal stem cell function. This combination therapy prevented bone loss in postmenopausal osteoporosis models and restored osteoporotic bone regeneration [132]. Additionally, QCT attenuated bone loss in ovariectomized rats by modulating the inflammatory signaling pathway of gut flora-short chain fatty acids. It increased probiotic bacteria and reduced pathogenic bacteria, thereby increasing bone resistance and preventing ovariectomized-induced bone loss [124].

In osteoclastogenesis and bone resorption, Córdoba and colleagues (2018) evaluated the effect of QCT-coated titanium implants on osteoclast activity in vitro and in vivo. This approach significantly reduced osteoclastogenesis by decreasing the expression of osteoclast-related genes including RANKL [133]. In diabetic rats, it demonstrated antioxidant, antidiabetic, and osteoprotective effects by inhibiting osteoclastogenesis and modulating the Wnt/β-catenin and RUNX2 pathways [134]. Regarding bone healing and regeneration, Durmaz et al. (2023) found that QCT increased parameters related to bone consolidation in critical-sized tibial defects in rats. When combined with xenografts QCT increased the healing and ossification rates, suggesting its potential use as an adjunct to grafting materials to promote bone repair [135]. No less important, a calcium sulfate hemihydrate/nano-hydroxyapatite compound enriched with QCT was developed for bone repair in critical tibial defects. This formulation demonstrated good biocompatibility, optimized osteogenic potential and enhanced proliferation, migration, and osteogenic differentiation of BMSCs. Moreover, it exhibited in vivo anti-inflammatory effects, suggesting superior efficacy in facilitating bone repair [136].

Complementing these findings, 4-methylcatechol, a metabolite of QCT, exhibits potent anti-inflammatory and antioxidant properties with promising applications for rheumatoid arthritis. This compound promotes macrophage polarization toward the M2 phenotype while inhibiting M1 polarization and suppressing pyroptosis. By modulating key signaling pathways, such as NRF2/HO-1 and NF-κβ/NLRP3, 4-methylcatechol expands its therapeutic potential to inflammatory conditions, further underscoring the clinical relevance of QCT-derived compounds in both bone repair and immune regulation [77].

### 6.4. Ferulic Acid

Ferulic acid (FA) is a derivative of cinnamic acid and a member of the phenylpropanoid family [137]. Its structure consists of methoxy and hydroxy substituents containing trans-cinnamic acid at positions 3 and 4, respectively, on the phenyl ring. Its chemical formula is C_10_H_10_O_4_ with a molecular weight of 194.18 g/mol. It is commonly found in the ethanolic extracts of Brazilian brown and green propolis at different concentrations [24] along with Chinese propolis [3], which is sourced from commelinid plants, including grains such as rice, wheat, and oats, as well as in vegetables, fruits and nuts [138].

Doss et al. (2018) demonstrated the ability of FA to suppress osteoclast differentiation and prevent bone erosion through its inhibition of the RANKL-dependent NF-κβ signaling pathway. This study highlighted the potential for FA to mitigate pathological bone loss associated with conditions such as rheumatoid arthritis. The findings revealed a significant attenuation of RANKL-induced osteoclast differentiation, accompanied by decreased bone resorption activity and a downregulation of the NF-κβ signaling pathways [138].

Regarding osteoclast formation and function, Sagar and coworkers (2016) observed that FA effectively inhibited osteoclastogenesis from human peripheral blood CD14+ monocytes, disrupted actin ring formation, and reduced bone resorption activity. The study also revealed that FA suppressed the expression of dendritic cell-specific transmembrane proteins, a key regulator of osteoclast fusion, and induced apoptosis in mature osteoclasts through the caspase-3 pathway [139]. In exploring the protective effects of FA against total body irradiation (TBI)-induced bone marrow damage, Wagle et al. (2021) found that FA supplementation in mice mitigated TBI-induced bone mass loss, stem cell senescence, and hematopoietic defects. Their research highlighted FA’s role in enhancing antioxidant defenses, reducing ROS accumulation, and suppressing osteoclastic activation, thus promoting osteogenic activity [140].

Another report by Hou et al. (2019) focused on the protective effects of FA against glucocorticoid-induced osteoporosis in neonatal rats [141]. Their findings indicated that FA treatment increased bone mineral density and improved bone mechanical properties, likely through the activation of Sirtuin-1 and NF-κβ pathways, suggesting its potential as a therapeutic agent for glucocorticoid-induced osteoporosis [141]. In this sense, Zhou and colleagues (2021) investigated the effects of FA on osteoblast proliferation and oxidative stress in the context of glucocorticoid-induced osteoporosis. Their findings demonstrated that FA improved osteoblast proliferation and alleviated oxidative stress by modulating the ERK signaling pathway [142].

Du et al. (2017) investigated the role of FA in promoting osteogenesis in BMSC, demonstrating that FA enhances β-catenin expression by inhibiting microRNA-340 through hypoxia-related mechanisms, thereby supporting osteogenic differentiation. In a complementary study, Du et al. (2021) explored FA’s effects on IL-1β-induced chondrocyte degeneration in osteoarthritis, revealing that this compound activates the Sirtuin 1/Activated Protein Kinase/Peroxisome Proliferator-Activated Receptor Gamma Coactivator 1-Alpha signaling pathway to mitigate chondrocyte degeneration by reducing inflammatory mediators and oxidative stress, positioning FA as a promising therapeutic agent for osteoarthritis [143,144].

FA’s role in promoting bone defect repair after radiation exposure was also examined [145]. The results suggest that FA preserves the stemness of skeletal stem cells and reverses radiation-induced damage by activating the p38/MAPK and ERK/MAPK pathways. In a distinct context, Chai et al. (2023) investigated the reparative effects of a platelet-rich plasma/FA hydrogel compound in treating degenerative discs in rats. Their results showed that this compound promoted extracellular matrix synthesis and strengthened degenerative intervertebral discs, suggesting a novel therapeutic approach for intervertebral disc degeneration [146]. When copper(II) and zinc(II) FA complexes were tested for bone formation [137], an enhancement of the expression of osteoblast markers, including RUNX2 and type I collagen, was observed, alongside the promotion of bone mineralization in zebrafish embryos and the improvement of bone healing in adult zebrafish scales. Bider and coworkers (2024) investigated the use of 3D bioprinted multifunctional hydrogels composed of dialdehyde alginate and gelatin incorporated with FA. The addition of FA enhanced antioxidant and antimicrobial properties, with the optimal concentration (0.15% FA) improving cell viability for pre-osteoblastic MC3T3-E1 cells, making it promising for bone tissue engineering. However, challenges, such as controlled FA release and optimizing the material’s effective modulus, were noted [147].

### 6.5. Pinocembrin

Pinocembrin is a bioactive flavonoid compound from propolis which is sourced from various sources, including honey [11,23] and plants like *Herba patriniae*, *Isatidis radix*, *Menthae herba*, and *Siphonostegiae herba*, among others [148]. Pinocembrin, also known as 5,7-dihydroxyflavanone, is a flavanone characterized by a single bond rather than a double bond between C2 and C3 and is in a reduced state compared to other flavonoids. Its chemical formula is C_15_H_12_O_4_ with a molecular weight of 256.25 g/mol.

Natsume et al. (2021) investigated the impact of Pinocembrin on osteoblast differentiation using MC3T3-E1 cells. Their results indicated that pinocembrin enhanced ALP activity and mineralization, along with an increased mRNA expression for ALP and Osteocalcin genes, also elevating the mRNA expression for RUNX2 and Osterix. The study identified the BMP signaling pathway and estrogen receptor as key mechanisms behind Pinocembrin’s action, suggesting its bone anabolic potential and utility in osteoporosis prevention and treatment [149].

The therapeutic potential of Pinocembrin in inhibiting osteoclast formation and bone resorption was explored in vivo in an ovariectomy-induced osteolytic murine model (OVX) and in vitro by bone marrow macrophages (BMMs) of the femur and tibia obtained from C57BL/6J mice. Pinocembrin disrupted the interaction between RANKL and RANK, resulting in a suppression of the MAPK and NF-κβ signaling pathways, culminating in reduced NFATc1 core translocation and ROS production, inhibiting osteoclast formation and bone resorption activity. These results indicate that this compound could be a promising candidate for combating osteoclast-related bone loss [11]. Additionally, its potential in reducing intervertebral disc degeneration both in vivo and in vitro has also been studied. Pinocembrin was found to protect endplate chondrocytes from apoptosis and degeneration induced by oxidative stress by activating the nuclear factor erythroid 2-related factor 2 pathway, inducing mitophagy, inhibiting ferroptosis, and promoting cell viability. These results suggest that Pinocembrin may be an effective treatment for intervertebral disc degeneration by mitigating oxidative imbalance and mitochondrial damage [150].

Regarding glucocorticoid-induced apoptosis in osteocytes, this compound was shown to reduce cell viability loss and apoptosis in MLO-Y4 osteocyte-like cells by activating autophagy through a suppression of the Phosphoinositide 3-Kinase/Protein Kinase B/Mechanistic Target of Rapamycin pathway. These findings indicate that Pinocembrin could be a potential natural agent for preventing and treating glucocorticoid-induced osteonecrosis and avascular necrosis of the femoral head [12]. In terms of arthritis symptom reduction, Ahmed and colleagues (2021) evaluated the anti-arthritic effect of Pinocembrin in mice with adjuvant-induced arthritis. Its treatment reduced arthritic symptoms, including edema, redness, and impaired movement. Pinocembrin interacted with the transcription factor Sox4 and modulated the expression of inflammation-related signaling molecules, such as TNF-α, NF-κβ, and COX-2, which suggests Pinocembrin may serve as a therapeutic agent for arthritis [151].

### 6.6. Kaempferol

Kaempferol is found in extracts of Chinese, Brazilian, and Korean propolis [22,23], which is sourced from plants, such as *Ginkgo biloba* and *Moringa oleifera* [152]. It is a tetrahydroxyflavone with four hydroxy groups located at the 3, 5, 7, and 4′ positions. Its chemical formula is C_15_H_10_O_6_ with a molecular weight of 286.24 g/mol. This naturally occurring flavonoid is found abundantly in vegetables, such as broccoli, apples, citrus fruits, strawberries, beans, and onions. [153].

Kaempferol promotes osteogenesis through several signaling pathways. Nie and colleagues (2020) demonstrated that this compound enhances the proliferation and osteogenesis of periodontal ligament stem cells by activating the Wnt/β-catenin signaling pathway, positively regulating the expression of osteogenic genes, such as ALP, RUNX2, Osterix, Osteocalcin, and β-catenin. Similarly, Sharma and Nam (2019) found that Kaempferol stimulates the Wnt/β-catenin signaling pathway in SaOS-2 osteoblasts (a human osteosarcoma cell line), increasing ALP activity, collagen synthesis, and the mRNA expression of RUNX2, Osterix, Osteopontin, and bone sialoprotein. Beyond the Wnt/β-catenin pathway, this compound also regulates other critical signaling pathways involved in osteogenesis. Gan et al. (2022) observed that Kaempferol promotes osteogenesis in BMSC, while H. Liu et al. (2021) reported that this compound enhances the osteogenic differentiation of BMSC and alleviates osteoporosis by downregulating miR-10a-3p and upregulating CXCL12, also known as stromal cell-derived factor-1 [154,155,156,157].

In addition to its osteogenic effects, Kaempferol also has anti-osteoclastogenic properties. It has been shown to inhibit autophagy, thereby activating apoptosis in murine macrophage cells, suggesting its potential role in treating bone metabolism disorders [158]. Yu et al. (2024) revealed that Kaempferol mitigates inflammatory bone loss induced by wear particles by inhibiting osteoclast differentiation and function through the downregulation of JNK and p38-MAPK signaling pathways [159]. Furthermore, Dong and colleagues (2024) experimentally validated the key targets and pathways of Kaempferol in osteoporosis treatment, showing a significant positive regulation of protein kinase B alpha expression and a negative regulation of matrix metalloproteinase-9 expression in MC3T3-E1 cells [160].

The efficacy of Kaempferol in treating osteoporosis and other bone conditions is supported by various therapeutic applications. Wang et al. (2022) investigated the osseointegration effect of micro-nanoimplants loaded with this compound in osteoporotic rats, showing that the Kaempferol micro-nanocomposite coating enhanced the osseointegration capacity of the implants. Ranjbar et al. (2023) developed bioactive glass-based scaffolds loaded with Kaempferol for bone tissue engineering, which demonstrated increased calcium deposition and ALP activity in vitro, along with complete bone regeneration in vivo. Network pharmacology and molecular docking studies have also explored Kaempferol’s mechanisms of action. Tang et al. (2022) identified that this compound binds to active pockets of key targets through various interactions, regulating biological processes such as inflammatory response, oxidative stress, and bone homeostasis [14,15,161].

### 6.7. p-Coumaric Acid

P-coumaric acid is an aromatic acid with a hydroxy substituent located in the C-4 position of the phenyl ring. It is the conjugate acid of a 4-coumarate. Its chemical formula is C_9_H_8_O_3_ and it has a molecular weight of 164.16 g/mol. P-coumaric acid is one of the major biologically active phenolic components of Brazilian green propolis, which is sourced from the plant *Baccharis dracunculiforia* [63].

P-Coumaric acid has emerged as a promising multifunctional therapeutic agent for treating various degenerative joint conditions, including temporomandibular joint osteoarthritis, rheumatoid arthritis, and osteosarcoma. Its antioxidant, anti-inflammatory properties, along with its ability to inhibit ferroptosis, osteoclastogenesis, and bone resorption, as well as stimulate bone growth, make p-Coumaric acid a viable natural option for the treatment of these diseases [16,17,18,162,163].

Guo et al. (2024) demonstrated that poly(p-coumaric) nanoparticles synthesized from this compound exhibited superior efficacy compared to hyaluronic acid in alleviating temporomandibular joint osteoarthritis. These nanoparticles showed antioxidant and anti-inflammatory properties, enhancing cell proliferation and matrix synthesis while reducing inflammation, oxidative stress, matrix degradation, and chondrocyte ferroptosis, significantly contributing to cartilage and subchondral bone repair. In a distinct context, Neog and Rasool (2018) explored the targeted delivery of mannosylated liposomes encapsulating p-coumaric acid to synovial macrophages in a rheumatoid arthritis animal model, focusing on osteoclastogenesis and bone resorption. Their findings demonstrated that p-Coumaric acid inhibited osteoclast formation and bone resorption by promoting OPG production and preserving calcium content, highlighting its potential as a therapeutic agent for intervening in osteoclastogenesis [16,163]

Regarding the reduction of inflammation and cartilage erosion, Neog and coworkers (2017) examined the effects of p-Coumaric acid in a rat model of adjuvant-induced arthritis. This compound significantly suppressed inflammation, cartilage, and bone erosion by reducing inflammatory cytokine levels and osteoclastogenic factor expression. It also increased OPG expression, regulated the RANKL/OPG imbalance, and suppressed the expression of RANKL-induced transcription factors, demonstrating promising anti-arthritic effects [162]. In terms of bone growth stimulation, the effects of p-Coumaric acid were tested on the longitudinal growth of long bones in adolescent male rats [17]. This treatment significantly increased tibial length, growth plate height, and the expression of insulin-like growth factor 1, promoting cellular proliferation in the growth plate zones. Lastly, Yang and colleagues (2023) explored the effects of p-Coumaric acid on osteosarcoma cell growth. This compound effectively inhibited the proliferation, migration, and invasion of osteosarcoma cells and promoted apoptosis, exerting an anti-osteosarcoma effect by inhibiting of the Phosphoinositide 3-Kinase/Protein Kinase B signaling pathway [18].

### 6.8. Galangin

Galangin is a flavonol-like polyphenol with three hydroxyl groups on its carbon rings. It is converted in the liver into kaempferol and quercetin by cytochrome P450, both of which have antioxidant properties. Its chemical formula is C_15_H_10_O_5_ and it has a molecular weight of 270.24 g/mol. Galangin is a bioactive compound isolated from propolis [164], which is sourced from plants such as *Plantago major* L., *Alnus pendula Matsum*., and *Scutellaria galericulata* L. [165].

Studies suggest that Galangin holds significant therapeutic potential due to its antitumor and anti-inflammatory properties [19,166]. This compound acts by inhibiting critical signaling pathways, promoting cellular differentiation, and mitigating conditions such as osteosarcoma and glucocorticoid-induced osteoporosis. These findings highlight the potential of Galangin as a novel therapy for malignant and inflammatory bone diseases and highlight its value in oncology research [20,166,167].

Yang et al. (2017) examined the effects of this compound in human osteosarcoma cells, focusing specifically on the MG63 and U20S cell lines. Their results demonstrated that Galangin significantly reduces cell proliferation and accelerates apoptosis in MG63 cells, also inhibiting their migration and invasion. Furthermore, Galangin downregulates phosphoinositide 3-kinase and phosphorylation of threonine 308 on the protein kinase B expression, as well as cyclin D1 and matrix metalloproteinases 2/9. Additionally, it upregulates p27Kip1, caspase-3, and caspase-8 levels, indicating that Galangin may suppress osteosarcoma cells via an inhibition of the Phosphoinositide 3-Kinase/Protein Kinase B pathway [166].

The effects of this compound on BMSC under inflammatory stress induced by lipopolysaccharides were investigated by Wang and Xiao (2023). Galangin increased cell viability and suppressed apoptosis, while reducing the levels of TNF-α, IL-1β, and IL-6 by downregulating NF-κβ phosphorylation. Additionally, this compound enhanced osteogenic differentiation by upregulating the expression of osteogenic markers, such as collagen type I alpha 1 chain, osteopontin, and RUNX2 [19].

Regarding glucocorticoid-induced osteoporosis, Zeng and colleagues (2023) explored the therapeutic effects of Galangin in dexamethasone-treated mice. Their study showed that this compound significantly alleviated the severity of osteoporosis. It enhanced the mineralization of BMSC by promoting autophagic flux through the Protein Kinase A/cAMP Response Element-Binding Protein pathway, suggesting its potential as a therapeutic agent for treating glucocorticoid-related osteoporosis [20]. In a study on osteoclastogenesis, Li et al. (2021) found that Galangin suppressed RANK by inhibiting the NF-κβ signaling pathway. This inhibition reduced the expression of specific osteoclast genes and suppressed bone resorption [167]. In summary, Galangin is a potential therapeutic agent for bone malignancies and inflammatory diseases due to its ability to inhibit key signaling pathways, promote osteogenic differentiation, and counteract bone degradation. These findings provide a solid foundation for further research into its clinical applications.

## 7. Summary of Bioactive Compounds from Propolis Extract on Bone Health: Future Direction and Final Remarks

**Figure 2:**
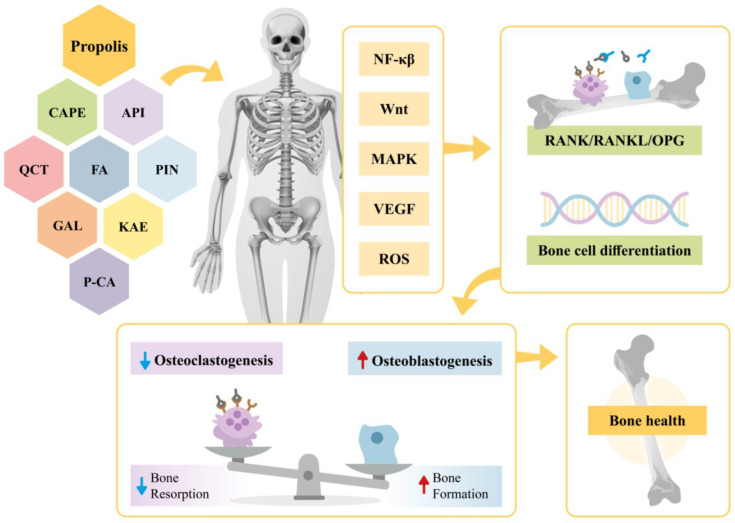
Effects of propolis and its bioactive compounds (CAPE, API, QCT, FA, PIN, KAE, P-CA, GAL) on bone metabolism. Propolis modulates signaling pathways (NF-κβ, Wnt, MAPK, VEGF, and ROS), influencing the RANK/RANKL/OPG system and promoting bone cell differentiation. This regulation reduces osteoclastogenesis, enhances osteoblastogenesis, and restores bone homeostasis. CAPE—Caffeic Acid Phenethyl Ester; API—Apigenin; QCT—Quercetin; FA—Ferulic Acid; PIN—Pinocembin; KAE—Kaempferol; P-CA—P-coumaric acid; GAL—Galangin; NF-κβ—Nuclear Factor kappa β; Wnt—Wingless/Integrated; MAPK—Mitogen-Activated Protein Kinase; VEGF—Vascular Endothelial Growth Factor; ROS—Reactive Oxygen Species; RANK/RANKL/OPG—Receptor Activator of Nuclear Factor κβ-Receptor Activator of Nuclear Factor κβ Ligand-Osteoprotegerin.

This narrative review provides evidence on the effects of propolis extract on bone health. Specifically, it highlights the impacts of propolis bioactive compounds in various contexts, including their roles in signaling pathways, osteogenesis, and angiogenesis as well as their antioxidant and anti-inflammatory actions. A summary of these findings is illustrated in Figure 2.

In summary, the therapeutic potential of propolis extract and its bioactive compounds for bone health is well-supported by current data. However, the future prospects for research on propolis and bone health are promising and multifaceted. The discovery of novel bioactive compounds in propolis continues to be an area of active investigation, with the potential to identify previously unknown molecules beneficial to bone health. Furthermore, in-depth studies on the mechanisms of action and molecular pathways of already known bioactive compounds will be crucial to fully elucidating how these molecules influence bone metabolism. Additional research is needed to clarify the specific interactions of these compounds with cell signaling pathways involved in bone formation and resorption, such as the RANKL/RANK/OPG, Wnt/β-catenin, and MAPK pathways. Moreover, investigations into the bioavailability, pharmacokinetics, and pharmacodynamics of these compounds in vivo are crucial for optimizing their therapeutic efficacy. Exploring the synergistic potential between drugs and bioactive compounds in propolis also represents a promising area of research, potentially leading to the development of more effective combination therapies for bone disorders.

## Data Availability

Not applicable.
